# High Prevalence of Left Ventricle Diastolic Dysfunction in Severe COPD Associated with A Low Exercise Capacity: A Cross-Sectional Study

**DOI:** 10.1371/journal.pone.0068034

**Published:** 2013-06-27

**Authors:** Marta López-Sánchez, Mariana Muñoz-Esquerre, Daniel Huertas, José Gonzalez-Costello, Jesús Ribas, Federico Manresa, Jordi Dorca, Salud Santos

**Affiliations:** 1 Department of Pulmonary Medicine, Hospital Universitari de Bellvitge, Barcelona, Spain; 2 Department of Cardiology, Hospital Universitari de Bellvitge, Barcelona, Spain; 3 Pneumology Research Group, Institut d´Investigacions Biomèdiques de Bellvitge (IDIBELL), Universitat de Barcelona, Barcelona, Spain; Pulmonary Research Institute at LungClinic Grosshansdorf, United States of America

## Abstract

**Background:**

A subclinical left ventricle diastolic dysfunction (LVDD) has been described in patients with chronic obstructive pulmonary disease (COPD).

**Objectives:**

To evaluate the prevalence of LVDD in stable severe COPD patients, to analyze its relationship with exercise capacity and to look for its possible causes (lung hyperinflation, ventricular interdependence or inflammatory mechanisms).

**Methods:**

We evaluated 106 consecutive outpatients with severe COPD (FEV_1_ between 30–50%). Thirty-three (31%) were excluded because of previous heart disease. A pulmonary function test, a 6-minute walking test (6MWT), a Doppler echocardiography test, including diastolic dysfunction parameters, and an analysis of arterial blood gases, NT-proBNP and serum inflammatory markers (CRP, leucocytes), were performed in all patients.

**Results:**

The prevalence of LVDD in severe stable COPD patients was 90% (80% type I, n=57, and 10% type II, n=7). A significant association between a lower E/A ratio (higher LVDD type I) and a lower exercise tolerance (6-minute walked distance (6MWD)) was found (r=0.29, p<0.05). The fully adjusted multivariable linear regression model demonstrated that a lower E/A ratio, a DLCO in the quartile 4^th^ and a higher tobacco consumption were associated with a lower 6MWD (76, 57 and 0.7 metres, respectively, p<0.05). A significant correlation between E/A ratio and PaO_2_ was observed (r=0.26, p<0.05), but not with static lung hyperinflation, inflammation or right ventricle overload parameters.

**Conclusion:**

In stable severe COPD patients, the prevalence of LVDD is high and this condition might contribute in their lower exercise tolerance. Hypoxemia could have a concomitant role in their pathogenesis.

## Introduction

Chronic obstructive pulmonary disease (COPD) is associated with relevant extrapulmonary effects and comorbidities that may influence the course of the disease [1]. Cardiovascular disorders are among the most prevalent. In fact, COPD is considered an independent cardiovascular risk factor [2, 3] and ischemic heart disease is one of the main causes of mortality in COPD [4]. Coexistence of both diseases is very common and has diagnostic, therapeutic and prognostic implications [5,6].

In COPD, the airflow obstruction is the major factor limiting exercise tolerance, but patients with similar FEV_1_ often have different degrees of dyspnea and exercise capacity, meaning that there might be other factors involved. In a previous study conducted in elderly COPD patients, the prevalence of unrecognized heart failure was over 20%, and isolated diastolic heart failure was involved in half of these cases [7]. However, little is known about its accurate prevalence in systematic studies and its effect on the exercise capacity.

In severe emphysema, smaller cardiac cavities and lower left ventricular (LV) end-diastolic volumes (measured by Magnetic Resonance Imaging (MRI)) have been previously reported [8]. More recently, echocardiographic findings have demonstrated a subclinical LV filling impairment, specially associated with the lung static hyperinflation [9]. Moreover, LV volumes improved in patients with severe emphysema who had undergone lung volume reduction surgery [10,11]. All these evidences suggest that hyperinflation plays a role, at least in advanced stages of the disease. However, a relationship between LV diastolic filling and a percentage of emphysema has also been observed in patients with mild flow obstruction (without hyperinflation) [12]. This means that other pathogenetic causes of impaired LV filling should be investigated. Loss of vascular bed or inflammatory mechanisms associated with COPD are postulated as potential additional factors.

The aim of our study was to determine the prevalence of LVDD as assessed by Doppler echocardiography in a cohort of stable severe COPD patients. We also analyzed its impact on functional capacity and its relation to lung hyperinflation, right ventricular (RV) function or inflammatory parameters.

## Methods

### Subjects and Study Design

A cross-sectional study of a prospective cohort of consecutive patients with stable severe COPD, who were evaluated between March 2010 and May 2011 at the Pulmonary Department of Bellvitge University Hospital (Hospitalet de Llobregat, Barcelona, Spain), was performed. Severe COPD was defined as forced expiratory volume in one second (FEV_1_) between 30–50% of reference value, with a ratio of FEV_1_ to FVC (forced vital capacity) below 0.70, according to the established criteria of the Global Initiative for Chronic Obstructive Lung Disease guidelines (GOLD) [13]. For the purpose of our study, patients with a previous history of coronary heart disease, heart valve disease, symptomatic peripheral artery disease, atrial fibrillation or with a Charlson score of more than 5 were excluded. A Doppler echocardiogram, complete lung function tests, a 6-minute walking test (6MWT) and blood samples and arterial gases analysis were performed at stable stage. Patients were considered to be in stable stage when they did not require changes in their regular medication for at least six weeks. Clinical and demographic data, specifically cardiovascular risk factors, were collected. The study protocol was approved by our institution’s local ethics committee (“Comité Ètic d’Investigació Clínica de l’Hospital Universitari de Bellvitge”, number PR258/09) and written informed consent was obtained from every subject.

### Blood Samples

Routine tests were done for blood count, inflammatory markers (leukocytes and C-reactive protein (CRP)), N-terminal pro-B-type natriuretic peptide (NT-proBNP) and analysis of arterial blood gases.

### Pulmonary Function

Pre- and post-bronchodilator spirometry (performed 15 min after administration of 400 µg of inhaled salbutamol), static lung volumes by plethysmography and single-breath carbon monoxide diffusing capacity (DLCO) were measured according to ATS/ERS recommendations using established reference values [14].

### Cardiac Function

Transthoracic echocardiographic studies were performed using a standard device (Vivid 7, General Electrics, USA). Measurements were done at least twice by the same operator and were performed at rest and at end-expiration. Due to the inherent difficulty of obtaining a good image quality of these patients, two experienced sonographers reported the studies. All records were considered technically acceptable to measure diastolic dysfunction parameters.

Images comprising 2-dimensional views from parasternal long-axis, mid-ventricular short-axis, and two and four chambers views were collected. LV wall thickness, LV end-diastolic and end-systolic diameters, RV diameter and left and right atrium areas were measured according to current recommendations. Measurement of left ventricular ejection fraction (LVEF) was performed by M-mode echocardiography using the Teicholtz method, by quantitative 2-D (biplane Simpson) method, and by 2-D visually estimated method. Parameters obtained from Doppler analysis were: peak flow velocity of early diastolic filling (E wave), peak flow velocity of late atrial filling (A wave) (both with and without Valsalva maneuver), pulse-wave Doppler of pulmonary venous flow when available and septal and lateral annular tissue Doppler velocities (septal and lateral e’). Estimation of the systolic pulmonary artery pressure (sPAP) was done using the modified Bernoulli equation with an estimated right atrial pressure of 10 mmHg. Tricuspid annular plane systolic excursion (TAPSE) was collected as variable related to RV function.

LV diastolic function was assessed by the following variables: deceleration time of the early transmitral flow (DT), ratio of early (E) to late (A) transmitral filling velocities (E/A ratio) and changes with the Valsalva maneuver. It was classified as normal or diastolic dysfunction (DD) type I (impaired relaxation), type II (pseudonormal filling) or type III (restrictive filling), as previously described [15]. These guidelines were specifically used because all patients had a normal left atrial (LA) diameter. To better evaluate LV diastolic function and LV filling pressures, additional parameters obtained from tissue Doppler analysis, such as septal and lateral e´ and LV filling index (E/e’ ratio) were also considered. Diastolic function classification was a consensus of both sonographers.

### Functional Exercise Capacity

The 6MWT was conducted according to current guidelines [16]. Supplemental oxygen was used when patient had prior chronic respiratory failure with domiciliary therapy.

### Statistical Analysis

Data were expressed as mean ± SD or as median and interquartile range according to whether the variable followed a normal distribution (Kolmogorov-Smirnov test). Student t test or Mann–Whitney U test were used for comparisons of variables between groups, when appropriate. Categorical variables were expressed as frequencies and percentages. Correlations between variables were analyzed with Pearson’s coefficient. A p value of <0.05 was considered to be statistically significant.

Association between exercise tolerance (6MWD) and LVDD parameters (E/A ratio) were analyzed using multivariate linear regression models adjusted for potential covariates which can affect the exercise capacity, such as age, smoking history (pack years), PaO_2_, FEV_1_ or inspiratory-to-total lung capacity ratio (IC/TLC ratio). Data analysis was performed with the statistical SSPS software (version 18.0, Chicago, IL, USA).

## Results

### Overall Characteristics of the Patients

**Figure 1:**
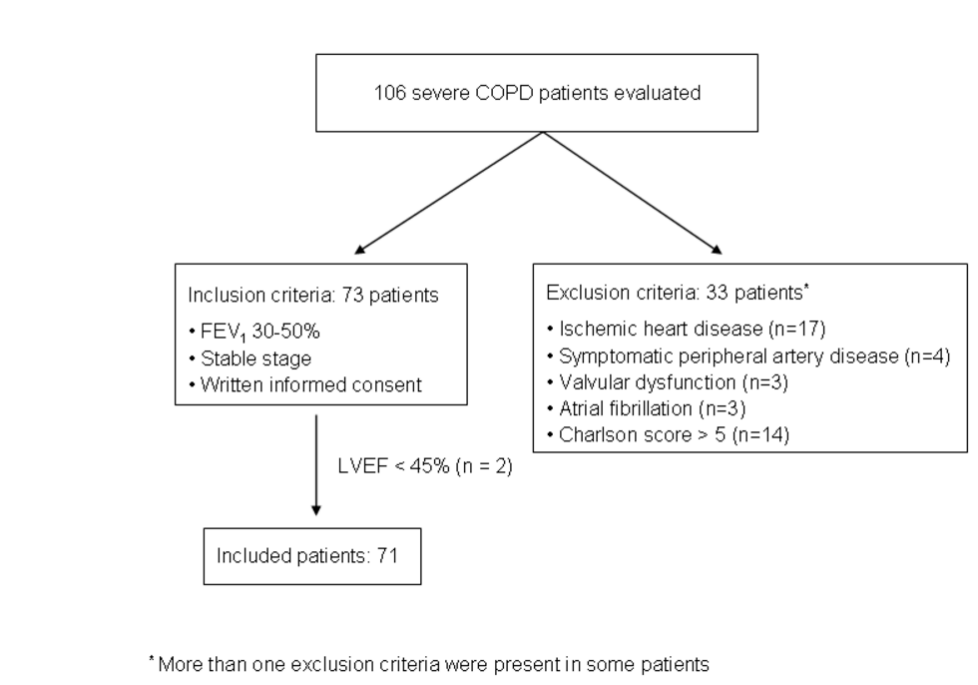
Patients evaluated during the study.

A total of 106 outpatients with stable severe COPD were evaluated during the study. Thirty-three patients (31%) were excluded because of previous heart disease. Two additional patients were also excluded because they presented unknown moderate LV systolic dysfunction, defined as LVEF < 45% [Figure 1].

The mean age of included patients was 65±7 years, and 91% (n=65) were male. All of them had been heavy smokers and 10% (n=7) continued to smoke. They had an elevated prevalence of cardiovascular risk factors. Table 1 shows the clinical characteristics of the study population.

**Table 1:** Patient characteristics.

Variables	All patients (n=71)	Non LVDD (n=7)		LVDD (n=64)
Age, years	65.5 ± 7.5	52.9 ± 9.3		66.9 ± 5.8*
Males, n (%)	65 (91.5)	5 (71.4)		60 (93.8)
BMI, Kg/m^2^	27.6 ± 5.1	24.0 ± 5.3		27.9 ± 5.0
Pack-years of smoking, n	57.8 ± 24.7	48.6 ± 22.1		58.8 ± 24.9
Current smokers, n (%)	7 (9.9)	2 (28.6)		5 (7.8)
Diabetes mellitus, n (%)	13 (18.3)			13 (20.3)
Dyslipidemia, n (%)	17 (23.9)			17 (23.9)
Systemic hypertension, n (%)	29 (40.8)	2 (28.6)		27 (42.2)
Use of statins, n (%)	13 (18.3)			13 (18.3)
Use of ACEIs or ARBs, n (%)	22 (31)	2 (28.6)		20 (31.3)

*Definition of abbreviations*: LVDD = Left ventricle diastolic dysfunction; BMI = body mass index; ACEI = angiotensin-converting enzyme inhibitor; ARA = angiotensin receptor antagonist. Values are expressed as mean±standard deviation or frequencies and percentages. *p < 0,05.

All patients were continuously treated with inhaled corticosteroids, long-acting beta-agonists and anticholinergics, and short-acting beta-agonists if needed. Twenty-two percent of patients (n=16) received an angiotensin-converting enzyme inhibitor and 8% (n=6) an angiotensin receptor antagonist. None of them received beta-blockers.

Lung functional and analytical characteristics are given in table 2. All these patients presented severe air trapping and static lung hyperinflation. The average 6MWD was 372±86 m and there was a wide range of BODE index (from 1 to 7). They had a mean PaO_2_ of 68±9 mmHg. However, 21% (n=15) of patients presented chronic respiratory failure and required long-term oxygen therapy. Mean analytical measures of NT-proBNP, CRP and other inflammatory markers were within the normal range.

**Table 2:** Lung functional and analytical characteristics.

Variables	All patients (n=71)	Non LVDD (n=7)	LVDD (n=64)
FVC, % pred	73.1 ± 14.9	85.9 ± 7.5	74.9 ± 15.1
FEV1, % pred	38.5 ± 6.1	36.9 ± 4.4	38.7 ± 6.3
TLC, % pred	127.4 ± 23.2	128.7 ± 15.2	127.2 ± 24.1
RV, % pred	216.4 ± 76.8	210.4 ± 38.7	217.1 ± 80.5
IC/TLC ratio	0.27 ± 0.07	0.30 ± 0.04	0.27 ± 0.07
DLCO/VA, % pred	63.2 ± 21.9	56.1 ± 27.2	64.1 ± 21.3
mMRC scale I–II/III, n (%)	54 (76) / 17 (24)	6 (85.7) / 1 (14.3)	48 (75) / 16 (25)
6MWD, m	372.1 ± 86.4	413.9 ± 43.2	367.5 ± 88.9
CRP, mg/L	8.3 ± 9.4	1.5 ± 0.8	8.9 ± 9.6*
NT-proBNP, ng/L	79.3 ± 70.4	64.3 ± 35.7	80.8 ± 72.9
PaO_2_, mmHg	68.4 ± 9.4	71.6 ± 12.3	68.1 ± 9.0
PaCO_2_, mmHg	44.1 ± 5.5	44.4 ± 5.8	43.9 ± 5.5

*Definition of abbreviations*: LVDD = left ventricle diastolic dysfunction; FVC = forced vital capacity; % pred = % predicted; FEV_1_ = forced expiratory volume in 1 second; TLC = total lung capacity; RV = residual volume; IC/TLC = inspiratory-to-total lung capacity ratio; DLCO/VA = lung diffusion capacity corrected for alveolar ventilation; mMRC = modified Medical Research Council dyspnea scale; 6MWD = 6-minute walking distance; CRP = C-reactive protein; NT-proBNP = N-terminal pro-B-type natriuretic peptide. Values are expressed as mean±standard deviation or frequencies and percentages. *p < 0,05.

### Cardiac Function

Table 3 shows the echocardiographic parameters. The LV wall thickness, systolic and diastolic diameters, the filling volume and the stroke volume tend to be low and the LA diameter was normal in the global study population. Although sPAP and maximal tricuspid regurgitation speed (V_max__._) average were in the normal range, 22% (n=16) of the patients presented echocardiographic criteria of pulmonary hypertension (PH) (sPAP> 40 mmHg and V_max__._ T> 2.8 m/s). Following classical criteria, the overall prevalence of LVDD was 90% (n=64) (80% type I and 10% type II). Only 7 patients (10%) presented normal DD echocardiographic parameters, so differences in clinical, functional and analytical characteristics between non LVDD and LVDD groups were not considered relevant in the analysis (Tables 2 and 3).

**Table 3:** Echocardiographic parameters.

Variables	Non LVDD (n=7)	LVDD Type I (n=57)	LVDD Type II (n=7)
Septal thickness, mm*	9 ± 2.5	11 ± 1.8	10.4 ± 1.7
Posterior wall, mm	9.8 ± 1.4	9.9 ± 1.7	9.6 ± 1.6
LVED diameter, mm*	48.8 ± 5.2	46.1 ± 6.9	52.9 ± 3.7
LVES diameter, mm*	29.8 ± 4.3	29.3 ± 5.2	34.1 ± 3.1
LA diameter, mm	35.8 ± 3	36.6 ± 6.7	38.3 ± 6.0
LVED volume, mL*	63.1 ± 17.5	78.3 ± 24.6	96.3 ± 20.2
LVES volume, mL*	21.2 ± 9	28.8 ± 11.9	36.3 ± 9.6
LV stroke volume, mL	42 ± 9	49.5 ± 14.9	60.0 ± 12.5
LVEF, %	68.1 ± 5.3	63.6 ± 6.8	61.3 ± 4.4
sPAP, mmHg	39.2 ± 8.1	38.7 ± 12.6	36.3 ± 11.4
TAPSE, mm	21.3 ± 3.7	21.2 ± 4.1	23.1 ± 2.3
Basal E/A ratio*	1.1 ± 0.2	0.7 ± 0.2	1.2 ± 0.1
Valsalva E/A ratio*	0.8 ± 0.1	0.6 ± 0.1	0.7 ± 0.2
E/e’ ratio	7.3 ± 1.9	8.7 ± 2.9	11.5 ± 4.9
DT, ms*	207.5 ± 65.4	257.2 ± 62.8	177.6 ± 38.7

*Definition of abbreviations*: LVDD = left ventricle diastolic dysfunction; LVED = left ventricular end-diastolic; LVES = left ventricular end-systolic; LA = left atrium; LVEF = left ventricular ejection fraction; sPAP = systolic pulmonary artery pressure; TAPSE = tricuspid annular plane systolic excursion; E = peak early mitral flow velocity, A = peak late mitral flow velocity; e’ = velocity of mitral annulus early diastolic motion; E/A ratio = ratio of early (E) to late (A) mitral flow peak velocities; E/e’ ratio = left ventricular filling index; DT = deceleration time of the early mitral flow. Values are expressed as mean±standard deviation. *p < 0.05 between non-LVDD and LVDD (type I and type II) groups.

**Figure 2:**
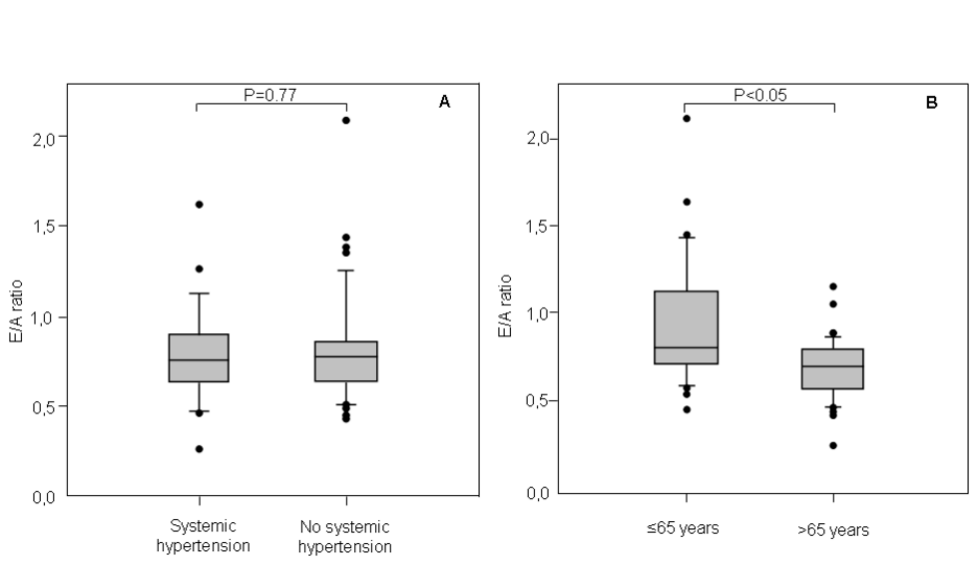
Individual data of the E/A ratio as measurement of the left ventricle diastolic dysfunction (LVDD). No significant differences were found in the E/A ratio between systemic hypertension and non-systemic hypertension patients (A). Although patients over 65 years had a significantly lower E/A ratio, younger patients also had a mean E/A ratio in the range of LVDD (B). Horizontal bars represent median values and box areas represent interquartile ranges. E/A ratio = ratio of early (E) to late (A) transmitral filling velocities.

The prevalence of LVDD between systemic hypertension and non-systemic hypertension patients was similar. Moreover, no significant differences were found in the degree of DD, measured as E/A ratio, between two groups [Figure 2, A]. Finally, although patients over 65 years had a significantly lower E/A ratio, younger patients also had a mean E/A ratio in the range of DD [Figure 2, B].

### Exercise Tolerance

**Figure 3:**
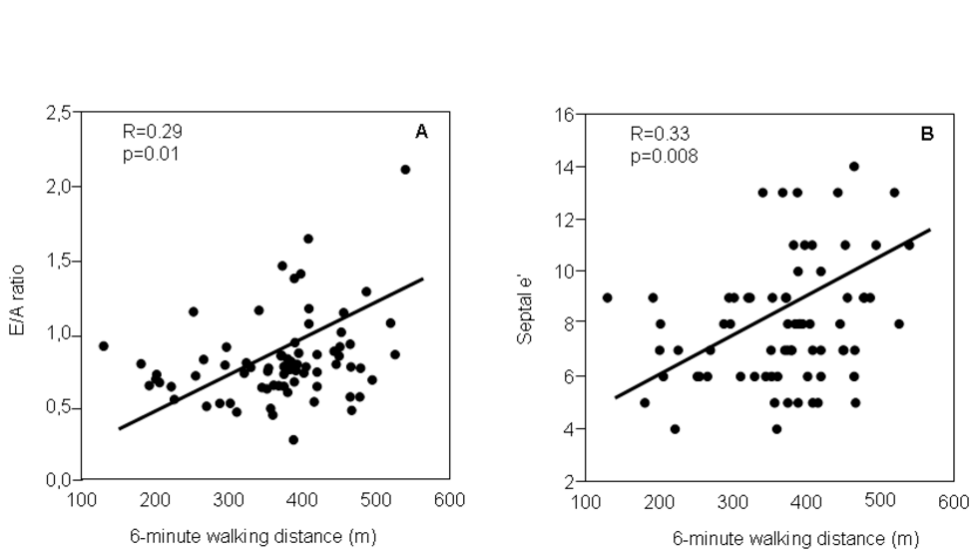
Relationship between the left ventricle diastolic dysfunction and the exercise tolerance in severe COPD patients. A lower 6-minute walking distance correlated with a lower E/A ratio (A) and a lower septal e’ (B). E/A ratio = ratio of early (E) to late (A) transmitral filling velocities.

A decreased walking distance in the 6MWT was associated with a lower E/A ratio (r=0.29, p <0.05) and a lower septal e´ (r=0.30, p<0.05) [Figure 3], both indicating a higher LVDD. Additionally, in the univariate analysis, the lower 6MWD was associated with lower DLCO (quartile 4^th^) and greater tobacco consumption [Table 4]. DLCO quartile 4^th^ is characteristic of more severe emphysema. In contrast, no association was found between 6MWD and other parameters that might have influenced the exercise tolerance in COPD, such as FEV_1,_ the presence of PH (sPAP) or lung hyperinflation (IC/TLC ratio).

**Table 4:** Univariate analysis of clinical, lung functional and echocardiographic variables according to the 6-minute walking distance (m).

Variables	Coefficient β (95% CI)	*p*-value
E/A ratio	88.2 (20.4 to 156.0)	0.012*†
PaO_2_, mmHg	1.9 (-0.4 to 4.2)	0.102†
DLCO quartile 4^th^	-63.2 (-110.1 to -16.3)	0.009*†
Smoking, pack-years	-0.9 (-1.7 to -0.1)	0.024*†
Age, years	-2.5 (-5.2 to 0.2)	0.071†
TAPSE, mm	5.0 (-0.2 to 10.2)	0.060†
FEV_1_, % pred	1.0 (-2.4 to 4.4)	0.557
IC/TLC ratio	3.7 (-336.2 to 343.6)	0.983
Diabetes	-24.2 (-77.2 to 28.7)	0.364
Pulmonary hypertension	10.3 (-37.1 to 57.6)	0.666
BMI, kg/m^2^	-0.13 (-4.2 to 3.9)	0.948
Use of ACEIs / ARBs	10.7 (-33.7 to 55.2)	0.632

*Definition of abbreviations*: CI = confidence interval; E/A ratio = ratio of early (E) to late (A) mitral flow peak velocities; DLCO = diffusing capacity for carbon monoxide; TAPSE = tricuspid annular plane systolic excursion; FEV_1_ = forced expiratory volume in 1 second IC/TLC = inspiratory-to-total lung capacity ratio; BMI = body mass index; ACEI = angiotensin-converting enzyme inhibitor; ARA = angiotensin receptor antagonist; * A p value <0.05 was considered to be statistically significant; †p<0.2 was considered for the multivariate analysis.

The fully adjusted multivariable linear regression model, with 6MWD as a dependent variable, demonstrated that for every 1 point fall in the E/A ratio, the 6MWD decreased 76m (p<0.05). In addition, a DLCO in the quartile 4^th^ was associated with a decrease of 57m in the 6MWD and, for each pack-year of tobacco consumption, the distance decreased 0.78m (p<0.05) [Table 5]. Age had no significant influence in any of the linear regression models.

**Table 5:** Multivariate analysis according to the 6-minute walking distance (m) as dependent variable.

Independent variables	Coefficient β (95% CI)	*p*-value
(Constant: 368, 20 m)		
E/A ratio	76,16 (12,1 to 140,2)	0.012*
Smoking, pack-years	-0,78 (-1,54 to -0,02)	0.043*
DLCO, quartile 4^th^	-57,05 (-101,3 to -12,8)	0.021*

*Definition of abbreviations*: CI = confidence interval; E/A ratio = ratio of early (E) to late (A) mitral flow peak velocities; DLCO = diffusing capacity for carbon monoxide. * A p value of <0.05 was considered to be statistically significant.

### Parameters related to left ventricular diastolic dysfunction

In our patients, no correlation was found between the degree of LVDD, as measured by E/A ratio, and levels of NT-proBNP or inflammatory blood markers (CRP, leukocytes), nor with sPAP or lung hyperinflation parameters. Instead, we found a significant correlation between LVDD, as measured by E/A ratio, and a lower PaO_2_ (r=0.27, p<0.05). Finally, associations with DLCO were consistent but not significant.

## Discussion

The main finding of our study was the high prevalence of LVDD (90%) in severe stable COPD patients. Although an association between increasing severity of airflow obstruction and decreased LV filling has already been described, to our knowledge this is the first study to analyze the prevalence of LVDD in a selected group of COPD patients with similar FEV_1_ and static lung hyperinflation. Moreover, the impaired LV diastolic filling and the reduced LV distensibility in these patients was independently associated with a reduced exercise tolerance as measured by the 6MWD.

Age and systemic hypertension are factors directly related to LVDD in the general population. In fact, the prevalence of mild LVDD in people older than 65 years with a normal LVEF is 21.7% and this increases to 27% in those older than 70 [17,18]. In our sample of severe COPD patients, classical echocardiographic parameters of LVDD were also observed regardless of age and lack of systemic hypertension. Furthermore, we didn’t find echocardiographic parameters of chronic systemic hypertension such as LV hypertrophy or LA dilation.

Echocardiography is the most usual and feasible non-invasive method to asses LV diastolic function. The E/A ratio mainly represent a measurement of LV filling. According to previous studies performed in COPD patients, a lower E/A ratio means an increased atrial contribution to this filling [19]. This occurs as well in the presence as in the absence of elevated PAP [20]. However, LVDD in COPD patients could be the result of decreased preload or pathological myocardial compliance. It is unknown which are the best echocardiographic parameters to measure LVDD. Evaluation of LV function in COPD patients may be improved using tissue Doppler echocardiography (TDE). One of the contributions of our study was to introduce tissue Doppler echocardiography (TDE) parameters, such as septal e´ and E/e´ratio, which are estimated measurements of LV wall compliance.

Previous studies have already shown that physical activity in patients with COPD is reduced [21] and that, in addition to the respiratory disturbances, it could be associated with impaired LV diastolic filling [22]. In our study, the exercise tolerance was measured by the distance walked in the 6MWT, which is a validated and reproducible test that shows the maximum sustainable exercise [23]. Moreover, the 6MWT is a good predictor of mortality in COPD [24]. For patients with severe disease, the 6MWT shows marked differences between individuals, so that the current multi-dimension indexes that include this parameter, such as BODE, are better predictors than the FEV_1_ which are used individually [25]. We demonstrate a meaningful association between the degree of LVDD, measured as E/A ratio and/or septal e’, and the exercise capacity as assessed by 6MWD, in patients with the same degree of COPD severity and significant hyperinflation. These results add importance to the cardiovascular status in the exercise capacity of severe COPD patients. Previous echocardiographic studies which address the LVDD and RV function in COPD and their relation with 6MWT present limitations, because patients with mild or moderate airflow obstruction have been selected [9,26,27]. Furthermore, some of the studies did not exclude other cardiovascular diseases and did not report the presence of systemic hypertension and comorbidities [26,27]. Sims et al [28] demonstrated a relationship between PH and a lower 6MWD in severe COPD patients. In contrast, our patients, with the same degree of severity, had lower exercise tolerance associated with higher LVDD, regardless of structural and functional changes of RV, measured by echocardiography.

Previous studies have shown that up to 31% of ICU admissions with a diagnosis of severe acute exacerbation of COPD might be of cardiac origin (associated with left heart dysfunction measured by echocardiography, most of the cases showing DD) and in these cases, the NT-proBNP is a serological marker that helps in clinical diagnosis [29]. BNP secretion might be secondary either to LV stress or to hypoxemia, as well as to PH or RV stress. In our stable COPD patients we did not find any relationship between the level of NT-proBNP and the degree of LVDD, hypoxemia or the presence of RV overload. So, it doesn’t seem to be a good marker of LVDD in non-acute stages of the disease.

Several mechanisms might explain the presence of LVDD in COPD patients. Watz et al. [9] described a significant correlation between LVDD and static pulmonary hyperinflation measured by the IC/TLC ratio in patients with different degrees of severity of COPD. Vassaux et al [30] showed an association between resting hyperinflation and lower oxygen pulse in patients with severe COPD, suggesting that a decreased cardiac function related to hyperinflation may contribute to exercise limitation. In our study, most patients had an IC/TLC ratio below 0.25. However, we found no significant correlation between IC/TLC ratio and echocardiographic parameters of LVDD, so hyperinflation might not be the only factor that determines LVDD in these patients.

In a high percentage of patients with advanced COPD, PH has already been described [31]. Abnormal patterns of LV diastolic filling have been reported in patients with increased RV afterload due to ventricular interdependence, either in idiopathic PH [32] than in PH associated with COPD [20,33,34]. Only 22% of our patients had echocardiographic criteria of PH (possibly underestimated), and we did not find any correlation between sPAP and LVDD parameters. However, endothelial dysfunction and vascular remodelling of pulmonary muscular arteries have been demonstrated in the entire disease spectrum, suggesting an impairment of pulmonary vessels distensibility and a subclinical increase in pulmonary vascular resistance [35,36]. These changes, together with loss of vascular bed due to emphysema, may develop decreased LV preload. In accordance with this hypothesis, a recent study has shown a link between LV filling volume, measured by MRI, and the degree of emphysema in patients with mild obstruction [12]. Interestingly, we observed a trend of association between the degree of LVDD and DLCO, a functional marker largely related to the degree of emphysema in COPD patients [37]. Some of the patients in our study (n = 43) had a computed tomography (CT) scan and, in these cases, we quantified the presence of emphysema (attenuation index lower than 950 HU) with a specific software. In the same way as Barr et al. [12] did in the general population, we also found that a higher emphysema index correlated with lower LV end-diastolic volume (R = -0.321, p <0.05) in severe COPD patients. In contrast, LVDD parameters did not change depending on the distribution of pulmonary emphysema.

According to our results, the severity of hypoxemia significantly correlates with the degree of LVDD in severe COPD patients. At advanced stages of the disease, hypoxemia is linked to the pathogenesis of pulmonary vascular abnormalities. In fact, Dinh-Xuan et al. [36] showed that hypoxemia is related to endothelial dysfunction. Moreover, chronic hypoxic pulmonary vasoconstriction might generate increasing pulmonary vascular resistance and a secondary decreased LV filling. Likewise, hypoxemia might also directly influence the cellular metabolism producing an impaired myocardial relaxation and higher LVDD [38].

Systemic inflammatory parameters that were measured in our patients (CRP, leucocytes) showed no association with LVDD measurements, so the inflammatory mechanism defining this disease [39,40] has not been demonstrated.

One of the strengths of the study was the homogeneous group selection of severe COPD patients. We excluded those with very severe COPD, since other limiting factors may influence their exercise capacity, and those with mild/moderate obstruction severity, because they often did not have any symptoms. This narrow selection avoids the influence of the degree of obstruction (FEV_1_) in the exercise tolerance.

Study limitations include the quality of echocardiography as a method to measure LVDD. Secondly, the study was performed at rest. Dynamic hyperinflation described in COPD during exercise may play a role in LVDD of these patients. Finally, our study was cross-sectional, so it is not possible to make firm conclusions about causality.

In summary, the prevalence of LVDD in patients with severe COPD is high, as assessed by standard echocardiographic measurements, even in the younger patients group and regardless of lack of systemic hypertension. These changes in LV volume filling and distensibility involve a decrease in exercise tolerance as measured by 6MWD. Besides static lung hyperinflation, other factors could be implicated in this condition. Loss of vascular bed due to emphysema, endothelial dysfunction or systemic parameters such as PO_2_ or inflammatory markers, also point to be causal mechanisms. Our study suggests that LVDD has clinical consequences in patients with severe COPD. However, further studies are needed to establish the causal relationship between LVDD and heart failure, one of the most prominent extrapulmonary manifestations of COPD.
