# Correction: High Prevalence of Left Ventricle Diastolic Dysfunction in Severe COPD Associated with A Low Exercise Capacity: A Cross-Sectional Study

**DOI:** 10.1371/annotation/b4120833-e4c6-42b5-92e9-24c396f9444e

**Published:** 2014-01-02

**Authors:** Marta López-Sánchez, Mariana Muñoz-Esquerre, Daniel Huertas, José Gonzalez-Costello, Jesús Ribas, Federico Manresa, Jordi Dorca, Salud Santos

The affiliation for the first author was incorrect. Marta López-Sánchez is not affiliated with #1,2 but with #1,3. The affiliation for the fourth author was also incorrect. José Gonzalez-Costello is not affiliated with #3 but with #2. The list of affiliation is as follows:

1. Department of Pulmonary Medicine, Hospital Universitari de Bellvitge, Barcelona, Spain

2. Department of Cardiology, Hospital Universitari de Bellvitge, Barcelona, Spain

3. Pneumology Research Group, Institut d´Investigacions Biomèdiques de Bellvitge (IDIBELL), Universitat de Barcelona, Barcelona, Spain

The Academic Editor's affiliation also contains an error. Henrik Watz is affiliated with the Pulmonary Research Institute at LungClinic Grosshansdorf, Germany, not the United States of America.

In Table 1, in the last row of the first column, "Use of ACEIs or ARBs" should be "Use of ACEIs or ARAs".

In Figure 3B, R=0.30, not .33.

